# High-risk additional chromosomal abnormalities at low blast counts herald death by CML

**DOI:** 10.1038/s41375-020-0826-9

**Published:** 2020-05-07

**Authors:** Rüdiger Hehlmann, Astghik Voskanyan, Michael Lauseker, Markus Pfirrmann, Lida Kalmanti, Sebastien Rinaldetti, Katharina Kohlbrenner, Claudia Haferlach, Brigitte Schlegelberger, Alice Fabarius, Wolfgang Seifarth, Birgit Spieß, Patrick Wuchter, Stefan Krause, Hans-Jochem Kolb, Andreas Neubauer, Dieter K. Hossfeld, Christoph Nerl, Alois Gratwohl, Gabriela M. Baerlocher, Andreas Burchert, Tim H. Brümmendorf, Jörg Hasford, Andreas Hochhaus, Susanne Saußele, Michele Baccarani, for the SAKK and the German CML Study Group, L. Fischer von Weikersthal, M. Hahn, G. Schlimok, D. Reichert, J. Janssen, U. Martens, P. Majunke, Peter Reichert, K. Neben, S. Korsten, Ch. Scholz, B. Oldenkott, J. Heßling, D. Kingreen, C. Sperling, C. Schelenz, I. Blau, A. Urmersbach, W. Ludwig, P. Le Coutre, R. Arnold, M. de Wit, A. Pezzutto, E. Schäfer, R. Schroers, A. Lochter, D. Behringer, Y. Ko, S. Weidenhöfer, W. Verbeek, P. Brossart, G. Trenn, W. Pommerien, J. Krauter, G. Doering, H. Munzinger, C. Diekmann, B. Hertenstein, S. Stier, F. Möller-Faßbender, M. Hänel, T. Zöller, C. Lamberti, B. Koch, A. Henzel, S. Wagner, A. Schmalenbach, M. Hoffknecht, G. Ehninger, A. Kiani, T. Illmer, C. Aul, M. Flaßhove, F. Henneke, M. Simon, L. Müller, H. Becker, R. Janz, M. J. Eckart, R. Fuchs, F. Schlegel, M. Wattad, R. Rudolph, D. W. Beelen, A. Lindemann, D. Linck, Wassman, E. Jäger, S. Al-Batran, T. Reiber, C. F. Waller, H. Hoeffkes, L. Schulz, K. Tajrobehkar, J. Mittermüller, H. Pralle, V. Runde, A. Hoyer, H. Tessen, L. Trümper, C. Schmidt, M. Sieber, H. Eschenburg, R. Depenbusch, S. Rösel, H. W. Lindemann, H. Wolf, C. Spohn, R. Moeller, D. Hossfeld, A. Zander, P. Schafhausen, H. Köster, W. Hollburg, N. Schmitz, H. Dürk, M. Hemeier, A. Grote-Metke, H. Weischer, B. Bechtel, L. Balleisen, M. Sosada, A. Ho, V. Petersen, J. Dengler, S. Bildat, L. Hahn, H. Dietzfelbinger, W. Gröschel, A. Bartholomäus, W. Freier, B. Sievers, I.-M. Pfreundschuh, T. Herrmann, A. Fauser, J. Menzel, M. Kemmerling, R. Hansen, H. Link, M. Schatz, M. Bentz, O. Prümmer, M. Kneba, J. Heymanns, S. Schmitz, C. Scheid, A. Lollert, M. Neise, M. Planker, M. Stauch, M. Schröder, B. Kempf, U. Vehling-Kaiser, S. Kremers, G. Köchling, L. Müller, F. Hartmann, T. Neuhaus, S. Fetscher, D. Kämpfe, G. Heil, M. Uppenkamp, B. Goldmann, T. Fischer Huber, U. Hieber, C. Plöger, M. Griesshammer, C. Lange, B. Göttler, C. Lunscken, X. Schiel, C. Scheidegger, O. Stötzer, H. Hitz, H. Schick, S. Völkl, K. Spiekermann, W. Berdel, H. Hebart, E. Ladda, P. Schmidt, U. Burkhardt, S. Hentschke, C. Falge, D. Reschke, C. A. Köhne, C. Müller-Naendrup, M. Sauer, S. Frühauf, K. Ranft, Y. Dencausse, B. Sandritter, G. Baake, M. Hofknecht, R. Dengler, M. Edinger, M. Schenk, A. Wehmeier, H.-P. Weidelich, R. Pihusch, K. Stahlhut, M. Baldus, A. Matzdorff, T. Geer, S. Schanz, G. Käfer, W. Gassmann, C. Priebe-Richter, M. Demandt, G. Springer, H. Fiechtner, C. Denzlinger, J. Schleicher, D. Assman, R. Gaeckler, G. Adam, A. Waladkhani, B. Rendenbach, H. Forstbauer, L. Kanz, S. Jacki, F. Stegelmann, N. Kalhori, A. Nusch, W. Langer, F. Müller, S. Brettner, B. Uebelmesser, T. Kamp, C. Schadeck-Gressel, K. Josten, O. Klein, R. Schwerdtfeger, H. Baurmann, H. Strotkötter, W. Fett, A. Raghavachar, C. Maintz, M. C. Goebler, R. Schlag, W. Elsel, M. Wernli, D. Heim, W. Wuillemin, U. Hess, J. Gmür, J. Mayer

**Affiliations:** 1 ELN Foundation, Weinheim, Germany; 2 III. Medizinische Klinik, Medizinische Fakultät Mannheim, Universität Heidelberg, Mannheim, Germany; 3 IBE Universität München, München, Germany; 4 MLL, München, Germany; 5 Institut für Humangenetik, MHH, Hannover, Germany; 6 Institut für Transfusionsmedizin und Immunologie, Medizinische Fakultät Mannheim, Universität Heidelberg und DRK-Blutspendedienst, Mannheim, Germany; 7 Medizinische Klinik 5, Universitätsklinikum, Erlangen, Germany; 8 Medizinische Klinik III, Universitätsklinikum Großhadern, München, Germany; 9 Klinik für Innere Medizin, Universitätsklinikum, Marburg, Germany; 10 2. Medizinische Klinik, Universitätsklinikum Eppendorf, Hamburg, Germany; 11 Klinikum Schwabing, München, Germany; 12 Universitätsspital, Basel, Switzerland; 13 Inselspital, Bern, Switzerland; 14 Uniklinik RWTH, Aachen, Germany; 15 Klinik für Innere Medizin II, Universitätsklinikum, Jena, Germany; 16 Department of Hematology-Oncology, Policlinico S.Orsola-Malpighi, University of Bologna, Bologna, Italy; 17 Klinikum St. Marien, Amberg, Germany; 18 Ambulantes Onkologiezentrum, Ansbach, Germany; 19 Klinikum, Augsburg, Germany; 20 Gemeinschaftspraxis für Hämatologie und Onkologie, Aurich, Germany; 21 Klinikum am Plattenwald, Bad Friedrichshall, Germany; 22 Klinikum, Bad Hersfeld, Germany; 23 Helios Klinikum, Bad Saarow, Germany; 24 Stadtklinik, Baden-Baden, Germany; 25 Vinzenz Pallotti Hospital Bensberg, Bergisch Gladbach, Germany; 26 Vivantes Krankenhaus Am Urban, Berlin, Germany; 27 St. Hedwig-Krankenhaus, Berlin, Germany; 28 Praxis für Innere Medizin, Berlin, Germany; 29 Onkologische Schwerpunktpraxis Tiergarten, Berlin, Germany; 30 Onkologische Schwerpunktpraxis Berlin-Mitte, Berlin, Germany; 31 Onkologische Praxis am Gesundbrunnen, Berlin, Germany; 32 MVZ Hämatologie Onkologie Tempelhof, Berlin, Germany; 33 Helios Klinikum Buch, Berlin, Germany; 34 Charité-Campus Virchow, Berlin, Germany; 35 Vivantes Klinikum Neukölln, Berlin, Germany; 36 Campus Benjamin Franklin, Berlin, Germany; 37 Onkologische Schwerpunktpraxis, Bielefeld, Germany; 38 Universitätsklinik Knappschaftskrankenhaus, Bochum, Germany; 39 Augusta-Kranken-Anstalt, Bochum, Germany; 40 Johanniter-Krankenhaus, Bonn, Germany; 41 Zentrum für ambulante Hematologie und Onkologie, Bonn, Germany; 42 Medizinische Klinik III, Bonn, Germany; 43 Knappschaftskrankenhaus, Bottrop, Germany; 44 Städtisches Klinikum, Brandenburg, Germany; 45 Klinikum, Braunschweig, Germany; 46 Gemeinschaftspraxis für Hämatologie und Onkologie, Bremen, Germany; 47 DIAKO, Bremen, Germany; 48 Klinikum Bremen Mitte, Bremen, Germany; 49 Onkologische Schwerpunktpraxis, Brühl, Germany; 50 Lukas Krankenhaus, Bünde, Germany; 51 Klinik für Innere Medizin 3, Chemnitz, Germany; 52 Onkologische Schwerpunktpraxis, Coburg, Germany; 53 Klinikum, Coburg, Germany; 54 St. Vincenz Krankenhaus, Datteln, Germany; 55 Krankenhaus Maria Hilf, Daun, Germany; 56 Klinikum, Deggendorf, Germany; 57 Städtische Kliniken, Delmenhorst, Germany; 58 Praxis für Hämatologie und Onkologie, Dernbach, Germany; 59 Universitätsklinikum Carl Gustav Carus, Dresden, Germany; 60 Gemeinschaftspraxis Hämatologie – Onkologie, Dresden, Germany; 61 HELIOS St. Johannes Klinik, Duisburg, Germany; 62 Krankenhaus, Düren, Germany; 63 Internistische Praxisgemeinschaft, Ehingen, Germany; 64 Onkologische Schwerpunktpraxis Leer, Emden, Germany; 65 Praxis für Innere Medizin, Hämatologie und Onkologie, Erkelenz, Germany; 66 Internistische Schwerpunktpraxis, Erlangen, Germany; 67 St. Antonius Hospital, Eschweiler, Germany; 68 Kliniken Süd, Essen, Germany; 69 Hämatologisch-Onkologische Gemeinschaftspraxis, Essen, Germany; 70 Klinik für Knochenmarktransplantation, Essen, Germany; 71 Praxis für Innere Medizin, Ettlingen, Germany; 72 Praxiskooperation, Euskirchen, Germany; 73 Universitätsklinikum, Frankfurt am Main, Germany; 74 Krankenhaus Nordwest, Frankfurt am Main, Germany; 75 Onkologische Praxis, Freiburg, Germany; 76 Universitätsklinikum, Freiburg, Germany; 77 Klinikum, Fulda, Germany; 78 Klinikum, Garmisch-Partenkirchen, Germany; 79 Praxis für Innere Medizin, Geilenkirchen, Germany; 80 Gemeinschaftspraxis für Hämatologie und Onkologie, Germering, Germany; 81 Universitätsklinikum, Gießen, Germany; 82 Wilhelm-Anton-Hospital, Goch, Germany; 83 Onkologische Kooperation Harz, Goslar, Germany; 84 Universitätsmedizin, Göttingen, Germany; 85 Universitätsklinikum, Greifswald, Germany; 86 Kreiskrankenhaus, Gummersbach, Germany; 87 Onkologische Schwerpunktpraxis, Güstrow, Germany; 88 Onkologische Schwerpunktpraxis, Gütersloh, Germany; 89 St. Marien-Hospital, Hagen, Germany; 90 Universitätsklinikum, Halle/Saale, Germany; 91 Hämatologisch – Onkologische Gemeinschaftspraxis, Halle/Saale, Germany; 92 Universitätsklinikum Eppendorf, Hamburg, Germany; 93 Hämatologisch-onkologisches Zentrum Ost, Hamburg, Germany; 94 Hämatologisch-onkologische Praxis Altona, Hamburg, Germany; 95 Allg. Krankenhaus St. Georg, Hamburg, Germany; 96 St. Marien-Hospital, Hamm, Germany; 97 Hämatologische Gemeinschaftspraxis, Hamm, Germany; 98 Evangelisches Krankenhaus, Hamm, Germany; 99 Klinikum Siloah, Hannover, Germany; 100 Universitätsklinikum, Heidelberg, Germany; 101 Praxis für Innere Medizin, Heidenheim, Germany; 102 Onkologische Schwerpunktpraxis, Heilbronn, Germany; 103 Medizinische Klinik II, Herford, Germany; 104 Praxisklinik, Herne, Germany; 105 Praxis für Hämatologie und Onkologie, Herrsching-Ammersee, Germany; 106 Onkologische Schwerpunktpraxis, Hersbruck, Germany; 107 St. Bernward Krankenhaus, Hildesheim, Germany; 108 Onkologie im Medicinum, Hildesheim, Germany; 109 Klinik für Innere Medizin, Homburg, Germany; 110 Klinikum, Idar-Oberstein, Germany; 111 Klinikum, Ingolstadt, Germany; 112 MVZ am Ev. Krankenhaus Bethanien, Iserlohn, Germany; 113 Hämatologisch-Onkologische Praxis, Kaiserslautern, Germany; 114 Westpfalzklinikum, Kaiserslautern, Germany; 115 St. Vincentius-Kliniken, Karlsruhe, Germany; 116 Städtisches Klinikum, Karlsruhe, Germany; 117 Klinikum Oberallgäu, Kempten, Germany; 118 Universitätsklinikum, Kiel, Germany; 119 Praxisklinik für Hematologie und Onkologie, Koblenz, Germany; 120 Praxis für Hämatologie und Internistische Onkologie, Köln, Germany; 121 Universitätsklinikum, Köln, Germany; 122 Onkologische Gemeinschaftspraxis, Krefeld, Germany; 123 Klinikum, Krefeld, Germany; 124 Onkologische Schwerpunktpraxis, Kronach, Germany; 125 Vinzentiuskrankenhaus, Landau, Germany; 126 Klinikum, Landshut, Germany; 127 Internistische Gemeinschaftspraxis, Landshut, Germany; 128 Caritas-Krankenhaus, Lebach, Germany; 129 Kreiskrankenhaus, Leer, Germany; 130 Onkologie UnterEms, Leer, Germany; 131 Klinikum, Lemgo, Germany; 132 St. Vincenz Krankenhaus, Limburg, Germany; 133 Sana Kliniken, Lübeck, Germany; 134 Praxis für Hämatologie/Onkologie, Lüdenscheid, Germany; 135 Klinikum, Lüdenscheid, Germany; 136 Klinikum Ludwigshafen, Ludwigshafen, Germany; 137 Onkologische Schwerpunktpraxis, Lüneburg, Germany; 138 Universitätsmedizin, Mainz, Germany; 139 Praxis für Innere Medizin, Mannheim, Germany; 140 Onkologie Praxis, Mannheim, Germany; 141 Johannes Wesling Klinikum, Minden, Germany; 142 Krankenhaus St. Franziskus, Mönchengladbach, Germany; 143 Onkologische Schwerpunktpraxis, Muhr am See, Germany; 144 Praxis für Innere Medizin, Mülheim, Germany; 145 Städtisches Klinikum Harlaching, München, Germany; 146 Praxis für Innere Medizin und HämatoOnkologie, München, Germany; 147 Onkologische Schwerpunktpraxis, München, Germany; 148 Hämatologische Schwerpunktpraxis, München, Germany; 149 Hämatologische Praxisgemeinschaft, München, Germany; 150 Onkologische Schwerpunktpraxis, München, Germany; 151 Universitätsklinikum Großhadern, München, Germany; 152 Universitätsklinikum, Münster, Germany; 153 Stauferklinikum Schwäbisch Gmünd, Mutlangen, Germany; 154 Onkologische Schwerpunktpraxis, Neumarkt, Germany; 155 Onkologische Schwerpunktpraxis, Neunkirchen, Germany; 156 Hämato-onkologisches Zentrum, Norderstedt, Germany; 157 Medizinische Klinik 5, Nürnberg, Germany; 158 Onkologische Praxis, Oldenburg, Germany; 159 Klinikum, Oldenburg, Germany; 160 Onkologische Schwerpunktpraxis, Olpe, Germany; 161 Martinus-Hospital, Olpe, Germany; 162 Paracelsus Klinik, Osnabrück, Germany; 163 Städtisches Krankenhaus, Penzberg, Germany; 164 MVZ am Siloah St. Trudpert Klinikum, Pforzheim, Germany; 165 Klinikum, Pforzheim, Germany; 166 Onkologische Praxis, Pinneberg, Germany; 167 Elisabeth Krankenhaus, Recklinghausen, Germany; 168 Schwerpunktpraxis für Hämatologie und Onkologie, Regensburg, Germany; 169 Universitätsklinikum, Regensburg, Germany; 170 Krankenhaus Barmherzige Brüder, Regensburg, Germany; 171 Sana Klinikum, Remscheid, Germany; 172 Klinikum am Steinenberg, Reutlingen, Germany; 173 Internistische Gemeinschaftspraxis, Rosenheim, Germany; 174 Immanuel Klinik, Rüdersdorf, Germany; 175 Internistische Schwerpunktpraxis, Rüsselsheim, Germany; 176 CaritasKlinikum St. Theresia, Saarbrücken, Germany; 177 Diakonie Hospital, Schwäbisch Hall, Germany; 178 Kreisklinikum, Siegen, Germany; 179 Kreiskrankenhaus, Sigmaringen, Germany; 180 St. Marien-Krankenhaus, Siegen, Germany; 181 Hämatologische und Onkologische Praxis, Stadthagen, Germany; 182 MVZ Onkologie am Klinikum Straubing, Straubing, Germany; 183 Praxis für Onkologie und Hämatologie, Stuttgart, Germany; 184 Marienhospital, Stuttgart, Germany; 185 Katharinenhospital, Stuttgart, Germany; 186 Diakonie-Klinikum Stuttgart, Stuttgart, Germany; 187 Bürgerhospital, Stuttgart, Germany; 188 Asklepios Klinik, Triberg, Germany; 189 Klinikum Mutterhaus der Borromäerinnen, Trier, Germany; 190 Internistische Gemeinschaftspraxis, Trier, Germany; 191 Hämatologisch-Onkologische Schwerpunktpraxis, Troisdorf, Germany; 192 Universitätsklinikum, Tübingen, Germany; 193 Internistische Praxis, Tübingen, Germany; 194 Universitätsklinikum, Ulm, Germany; 195 Gemeinschaftspraxis für Hämatologie und Onkologie, Velbert, Germany; 196 Praxis für Innere Medizin, Verden, Germany; 197 Kreiskrankenhaus, Waldbröl, Germany; 198 Hämatologische Praxis, Weiden, Germany; 199 Praxis für Innere Medizin, Wendlingen, Germany; 200 Schwerpunktpraxis für Hämatologie/Onkologie, Wesel, Germany; 201 Praxis für Hämatologie und Onkologie, Wiesbaden, Germany; 202 Deutsche Klinik für Diagnostik, Wiesbaden, Germany; 203 Praxis für Hämatologie und internistische Onkologie, Wuppertal, Germany; 204 Klinikum, Wuppertal, Germany; 205 Hämatologisch-Onkologische Praxis, Würselen, Germany; 206 Universitätsklinikum, Würzburg, Germany; 207 Hematologisch-Onkologische Schwerpunktpraxis, Würzburg, Germany; 208 Praxis für Innere Medizin, Zwickau, Germany; 209 Kantonsspital, Aarau, Switzerland; 210 Universitätsspital, Basel, Switzerland; 211 Kantonsspital, Luzern, Switzerland; 212 Kantonsspital, St. Gallen, Switzerland; 213 Onkozentrum Klinik im Park, Zürich, Switzerland; 214 Masaryk University Hospital, Brno, Czech Republic

**Keywords:** Translational research, Genetics research

## Abstract

Blast crisis is one of the remaining challenges in chronic myeloid leukemia (CML). Whether additional chromosomal abnormalities (ACAs) enable an earlier recognition of imminent blastic proliferation and a timelier change of treatment is unknown. One thousand five hundred and ten imatinib-treated patients with Philadelphia-chromosome-positive (Ph+) CML randomized in CML-study IV were analyzed for ACA/Ph+ and blast increase. By impact on survival, ACAs were grouped into high risk (+8, +Ph, i(17q), +17, +19, +21, 3q26.2, 11q23, −7/7q abnormalities; complex) and low risk (all other). The presence of high- and low-risk ACAs was linked to six cohorts with different blast levels (1%, 5%, 10%, 15%, 20%, and 30%) in a Cox model. One hundred and twenty-three patients displayed ACA/Ph+ (8.1%), 91 were high risk. At low blast levels (1–15%), high-risk ACA showed an increased hazard to die compared to no ACA (ratios: 3.65 in blood; 6.12 in marrow) in contrast to low-risk ACA. No effect was observed at blast levels of 20–30%. Sixty-three patients with high-risk ACA (69%) died (*n* = 37) or were alive after progression or progression-related transplantation (*n* = 26). High-risk ACA at low blast counts identify end-phase CML earlier than current diagnostic systems. Mortality was lower with earlier treatment. Cytogenetic monitoring is indicated when signs of progression surface or response to therapy is unsatisfactory.

## Introduction

Blast crisis (BC) of Philadelphia-chromosome-positive (Ph+) and/or BCR-ABL1-positive chronic myeloid leukemia (CML) is one of the remaining challenges in the management of the disease. Cytogenetic abnormalities and blast increase represent the most consistent indicators of progression to end-phase CML [1–7]. End-phase CML comprises early progression with emerging high-risk ACA and late progression with failing hematopoiesis and blast cell proliferation. BC represents the end stage of this evolution. Not all patients dying of CML reach the blast levels defining BC (20% or 30% blasts in blood or marrow) [8–11]. Once BC has occurred, treatment results are poor. Early allogeneic stem cell transplantation (SCT) might improve prognosis [12, 13]. Whether cytogenetic aberrations allow a timelier change of treatment with better outcome is unknown.

Additional chromosomal abnormalities (ACAs) are thought to result from BCR-ABL1-induced genetic instability and may be causative factors of disease progression [14, 15]. The most frequent ACAs found in BC (+8, a second Ph-chromosome (+Ph), an isochromosome of the long arm of chromosome 17, i.e., i[17q], and +19) were termed major route by Mitelman et al. [1–2]. Major-route ACAs have been associated with shorter survival, if they were detected at diagnosis [16] or if they emerged in the course of disease [17]. A poor prognosis was also observed with 3q26.2 and 11q23 rearrangements and with −7/7q− [18, 19], whereas +8 and +Ph as single aberrations, but not in combination, were not equally associated with poor prognosis [20]. Wang et al. [17] proposed a risk stratification of the six most frequent ACAs into two groups with distinct prognoses (+8, +Ph, −Y with good prognoses and i[17q], −7/7q−, 3q26.2 rearrangements with poor prognoses). Using the same cohort of patients, a risk stratification into three groups was proposed [21] based on BC risk associated with each ACA (high risk: 3q26.2; −7/7q−; i[17q]; complex karyotypes with high-risk ACA. Intermediate 1: +8; +Ph; other single ACA. Intermediate 2: other complex ACA).

Since the prognosis with single changes (+8, +Ph) is controversial, we decided to include these in our evaluation, as well as +21 and +17, which were designated as major route later on [1]. Other ACAs were not so clearly associated with shorter survival. This led to their tentative designation as low-risk ACA.

More recently, clonal chromosomal aberrations (CCAs) found in Ph-negative cells (CCA/Ph−) were reported to also have a negative impact on survival [22, 23].

We here made use of the data of 1551 imatinib-treated chronic phase (CP) patients recruited to CML-study IV, a randomized study comparing imatinib 400 mg with imatinib 800 mg and combinations of imatinib with interferon, simultaneously or sequentially, or low-dose cytarabine [24]. Our aim was to analyze if defined ACA at low blast levels allow an earlier diagnosis of end-phase CML and a timelier change of treatment than current blast thresholds.

## Patients and methods

### Patients

**Fig. 1:**
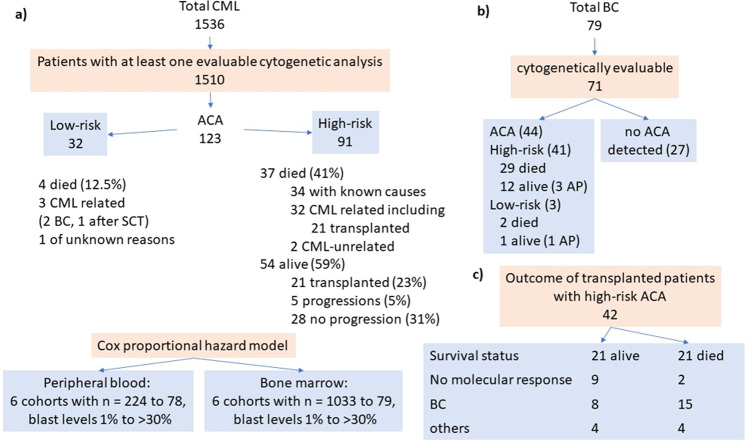
Flow chart. **a** Patients with ACA, **b** patients with BC, and **c** transplanted patients with high-risk ACA. BC blast crisis, AP accelerated phase, SCT stem cell transplantation.

Patient data were derived from the randomized CML-study IV (recruitment 2002–2012) with initial or predominant imatinib treatment [24]. Documentation was done at 3–6-month intervals as previously reported [24]. Risk assignment followed the ELTS (EUTOS Long-Term Survival) score [25]. BC was defined by 30% blasts in blood or marrow. Patient numbers and flow are depicted in the flow chart (Fig. 1a–c).

### Cytogenetics

By protocol, cytogenetic analysis was requested every 3–6 months during the early disease phases and every 12 months thereafter, if stable molecular remission (major molecular remission or better, BCR-ABL1 <0.1% on the International Scale, IS) was achieved. In stable molecular remission, intervals between cytogenetic analyses were frequently longer due to patients’ and/or doctors’ requests. A median of four analyses per patient were documented. Cytogenetic analyses were done as described and results were reported according to the international nomenclature (ISCN 2016) [16]. ACAs were evaluated if they were clonal according to the ISCN. Complex karyotypes were defined as three or more concurrent aberrations. High-risk ACAs were defined as the major route ACA +8, +Ph, i[17q], +19, +21, +17 (the ACA most frequently observed in BC) [1], the minor route ACA 3q26.2, 11q23, −7/7q− (less frequently observed, but negative impact on prognosis) [17, 19, 18], and complex karyotypes. Variant translocations and −Y were not considered, as they had no impact on prognosis in our and other studies [16, 26].

CAA/Ph− have not been an objective of this study.

### Molecular genetics

Molecular analyses followed the IS methodology and nomenclature [27–30].

### Statistics

For survival analyses, patients were followed up at the start of the diagnosis, at the time of the occurrence of an ACA, or at the time of a blast increase. Patients were censored at the date of last follow-up. Mortality after the advent of blast increases was assessed by Cox models starting at the time of a blast increase. Here the presence of ACA was considered as a time-dependent covariate. *P* values <5% were considered significant. Due to the explorative character of this work, no adjustment of *p* values was done and all *p* values have to be interpreted descriptively. All analyses were performed with SAS 9.3 or R 3.5.1.

## Results

### Identification of ACA

One thousand five hundred and thirty-six patients with Ph+ CP-CML were analyzed for blast increase and ACA, 1510 patients were cytogenetically evaluable. Patients’ characteristics are shown in Table 1. Median observation time was 8.6 years.

**Table 1:** Patient characteristics.

Patients (cytogenetically evaluable), *n*	1536 (1510)
Gender (%), male	60.2
Age at diagnosis of CML (years), median (range)	53 (16–88)
Hb (g/dl), median (range)	12.3 (4.7–19.1)
Platelets (×10^12^/μl), median (range)	375 (34–3020)
Patients with palpable splenomegaly	55.7%
ELTS-score (% low/intermediate/high)	57/30/13
WBC count at diagnosis (×10^9^/l) (median, range) with differential	76 (2.6–630)
Blasts (%)	1 (0–30)
Promyelocytes (%)	2 (0–34)
Basophils (%)	3 (0–66)
Median observation time (years)	8.6
10-Year survival	83%
Patients with ACA, high risk at diagnosis (*n*)	25
Patients with ACA, high risk in the course of disease (*n*)	66
High-risk ACA total (*n*)	91 (6%)
Patients with other-/low-risk ACA at diagnosis (*n*)	19
Patients with other-/low-risk ACA in the course of disease (*n*)	13
Low-risk ACA, total (*n*)	32 (2.1%)
ACA total (*n*)	123 (8.1%)
Patients with anemia (Hb < 10) at first appearance of ACA (%)	26.7^a^
Patients with thrombocytopenia (platelets < 75 × 10^9^/l) at first appearance of ACA (%)	17.8^a^
Patients with neutropenia (neutrophils < 1.5 × 10^6^/l) at first appearance of ACA (%)	15.5^a^
Patients with palpable splenomegaly (defined as spleen in cm below costal margin >0) at first appearance of ACA (%)	39.2^a^
Basophils at the time of ACA (%) (median, range)	1 (0–15)^a^
Age at diagnosis of ACA (years) (median, range)	52 (18–89)
Age at diagnosis of high-risk ACA (years) (median, range)	52 (23–89)
Median interval diagnosis—ACA (years) (median, range)	n.r. (0–11.1)^b^

^a^Only values up to 4 weeks in advance or 1 week after the first appearance of ACAs were counted (*n* ≥ 74).

^b^*Maximum number* = emergence of last ACA, *n.r.* not reported.

One hundred and twenty-three patients (8.1%) displayed ACA in Ph+ metaphases (Appendix A1). Ninety one (6%) were high-risk ACAs (+8, +Ph, i[17q], +17, +19, +21, 11q23 and 3q26.2 rearrangements, −7/7q abnormalities, complex karyotypes) and 32 (2.1%) were low-risk ACAs (all other). Of the 91 high-risk ACAs, 25 (1.7%) were detected at baseline and 66 (4.4%) emerged in the course of disease 0.5–133 months after diagnosis. The median time to detection of high-risk ACAs was 17 months. Of the 32 low-risk ACAs, 19 (1.3%) were detected at diagnosis and 13 (0.9%) emerged in the course of disease.

Frequencies of ACA are shown in Table 2 grouped according to type (risk level, single, or in combination) and time of emergence (at diagnosis or in the course of disease).

**Table 2:** Frequency of ACA.

Karyotypes	Single ACA	In combination with other ACA	Total
High-risk ACA (*n* = 91)^a^			
+8	19	19	38
At diagnosis	6	10	16
In the course of disease	13	9	22
+Ph	18	17	35
At diagnosis	7	6	13
In the course of disease	11	11	22
+19	0	11	11
At diagnosis	0	4	4
In the course of disease	0	7	7
+17/i(17q)	3	5	8
At diagnosis	1	3	4
In the course of disease	2	2	4
3q26.2	10	2	12
At diagnosis	1	0	1
In the course of disease	9	2	9
−7/7q abnormalities	5	4	9
At diagnosis	1	0	1
In the course of disease	4	4	8
+21	2	3	5
At diagnosis	1	1	2
In the course of disease	1	2	3
11q23	1	0	1
At diagnosis	0	0	0
In the course of disease	1	0	1
Complex karyotypes		25	25
At diagnosis		11	11
In the course of disease		14	14
Low-risk ACA	32	32	
At diagnosis	19	19	
In the course of disease	13	13	

^a^Multiple listings possible.

### Impact of ACA on survival

**Fig. 2:**
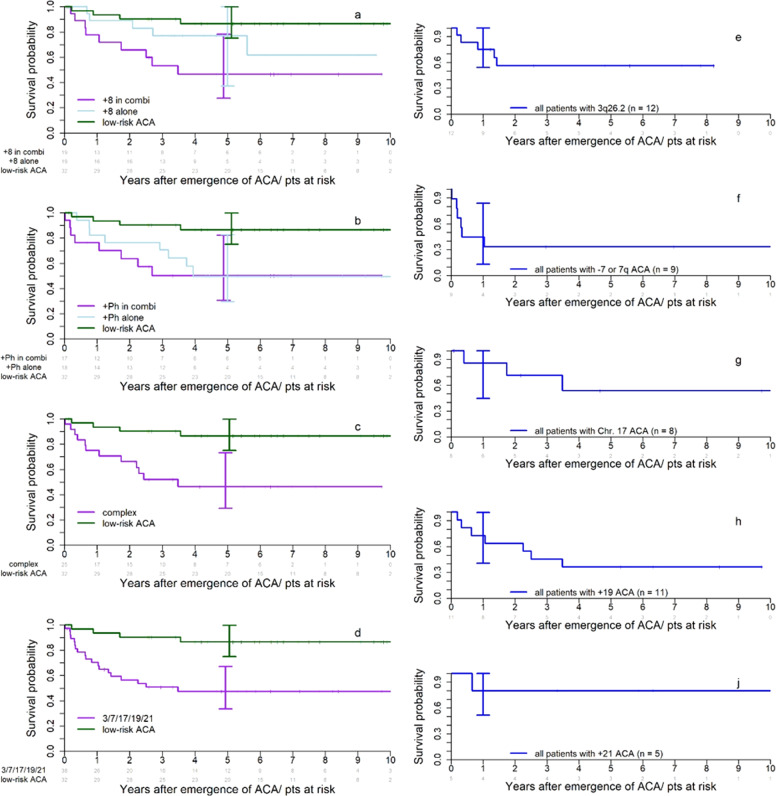
Impact of high- vs. low-risk ACA on survival. The left-side panel shows the impact of +8 (**a**), +Ph (**b**), complex ACA (**c**), and of chromosome 3, 7, 17, 19, and 21 aberrations combined (**d**) on survival in patients with primary imatinib treatment after the emergence of ACA. Suvival after emergence of low-risk ACA in imatinib-treated patients serves as control. The right-side panel shows the impact of rare high-risk ACA of chromosomes 3, 7, 17, 19, and 21 on survival (**e**–**i**).

Figure 2a–d shows the impact of high- and low-risk ACAs on survival from occurrence of ACA at diagnosis or in the course of disease. Observations were synchronized for the time of emergence of ACA, and ACA detected at diagnosis and emerging in the course of disease were analyzed together. All high-risk ACAs show a negative impact on survival compared to low-risk ACAs, which serve as control. An exception is +8 as a single aberration, which carries a prognosis in between high- and low-risk ACAs (Fig. 2a). Impact of +Ph on survival was equally poor whether it occurred alone or in combination with other abnormalities (Fig. 2b). Chromosome 3, 7, 17, 19, and 21 aberrations were grouped together, as they were rare (Fig. 2d). Individual analyses of these aberrations are shown in Fig. 2e–i. Four-year survival probability after occurrence of high-risk ACA, except +8 alone, was 52.2% (95% confidence interval (CI): 41–66), after occurrence of +8 alone 77% (95% CI: 60–100), and after occurrence of low-risk ACA 87% (95% CI: 75–100).

The treatment strategy after emergence of ACA including allogeneic SCT did not differ according to the type of ACA, but patients with high-risk ACA were transplanted more frequently. 42 of the 138 transplantations in CML-study IV (30%) were performed in the 6% of patients with high-risk ACA (Flow chart, Fig. 1c). Two-year survival of 26 patients transplanted in BC or accelerated phase (AP) was 46% (95% CI: 26–63%) and of 13 patients transplanted in CP 77% (95% CI: 44–92%; log-rank test: *p* = 0.09; phase unknown for 3 of 42 patients).

### Correlation of ACA with BC

79 patients developed BC during the observation time. Of the 79 BC patients, 71 were cytogenetically evaluable. 44 BC patients had ACA (61%), in 27 patients no ACAs were reported. Of the 44 BC patients with ACA, 41 (93%) had high-risk and 3 low-risk ACA (Flow chart, Fig. 1b).

### Correlation of ACA with blast increase

The close correlation of high-risk ACA with BC and the unfavorable prognosis of patients with high-risk ACA led us to ask if high-risk ACA can anticipate the diagnosis of end-phase CML. We therefore assigned patients, in whom a blast increase was observed in peripheral blood or marrow (at any time), to 6 different blast thresholds (1%, 5%, 10%, 15%, 20%, and 30%). In each of these cohorts high- and low-risk ACAs were considered as time-dependent variables. The number of patients ranged from 224 to 78 in the six cohorts with blast increases of 1% to >30% in the peripheral blood, and from 1033 to 79 in the six cohorts with blast levels of 1% to >30% in the marrow (Table 3). Naturally, the sets of patients who developed higher blast increases later on were subsets of the sets with lower blast increases.

**Table 3:** Hazard to die in IM-treated patients with high- and low-risk ACA dependent on blast increase to 1–30% (Cox model).

	HR	Lower 95% CI	Upper 95% CI	*P* value	*n*
Peripheral blood (PB)					
1% blasts in PB					
Presence of high-risk ACAs	3.65	2.32	5.75	<0.001	224
Presence of low-risk ACAs	1.92	1.06	8.07	0.039	
5% blasts in PB					
Presence of high-risk ACAs		1.11	2.86	0.016	117
Presence of low-risk ACAs	1.77	0.68	4.66	0.244	
10% blasts in PB					
Presence of high-risk ACAs	1.39	0.87	2.21	0.167	107
Presence of low-risk ACAs	1.38	0.53	3.60	0.506	
15% blasts in PB					
Presence of high-risk ACAs	1.37	0.86	2.19	0.189	104
Presence of low-risk ACAs	1.32	0.51	3.42	0.568	
20% blasts in PB					
Presence of high-risk ACAs	0.84	0.50	1.40	0.502	79
Presence of low-risk ACAs	0.74	0.20	2.71	0.652	
30% blasts in PB					
Presence of high-risk ACAs	0.83	0.50	1.39	0.479	78
Presence of low-risk ACAs	0.76	0.20	2.89	0.689	
Bone marrow (BM)					
1% blasts in BM					
Presence of high-risk ACAs	6.12	4.08	9.17	<0.001	1033
Presence of low-risk ACAs	2.71	0.99	7.44	0.053	
5% blasts in BM					
Presence of high-risk ACAs	5.46	3.58	8.33	<0.001	588
Presence of low-risk ACAs	3.21	1.16	8.85	0.024	
10% blasts in BM					
Presence of high-risk ACAs	2.21	1.37	3.56	0.001	134
Presence of low-risk ACAs	1.68	0.61	4.60	0.311	
15% blasts in BM					
Presence of high-risk ACAs	1.77	1.11	2.83	0.017	115
Presence of low-risk ACAs	1.66	0.63	4.37	0.309	
20% blasts in BM					
Presence of high-risk ACAs	1.24	0.74	2.06	0.416	87
Presence of low-risk ACAs	1.11	0.29	4.18	0.882	
30% blasts in BM					
Presence of high-risk ACAs	0.89	0.53	1.49	0.665	79
Presence of low-risk ACAs	0.81	0.21	3.07	0.760	

**Fig. 3:**
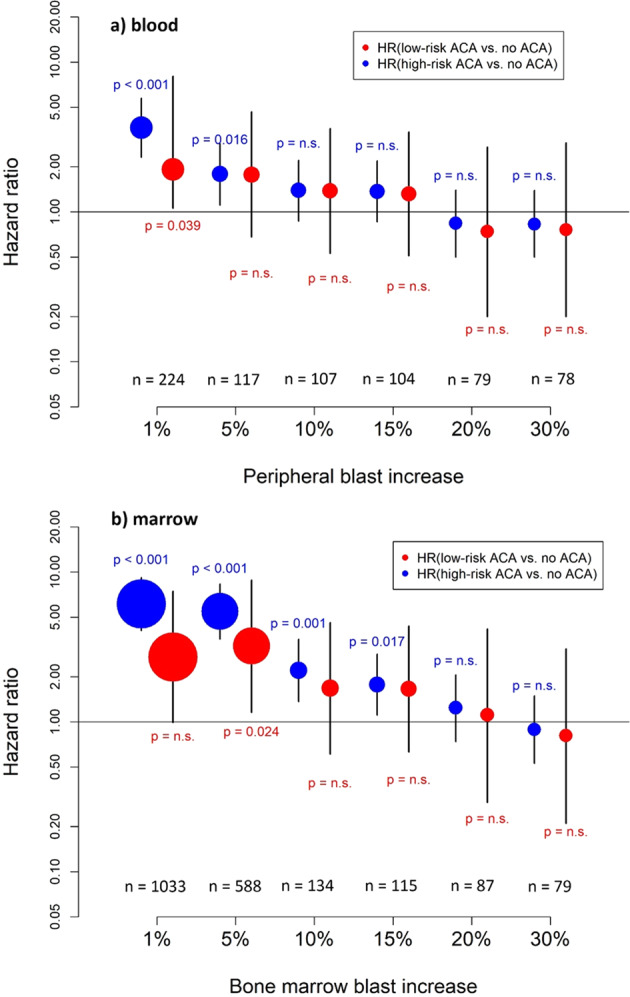
Hazard to die with high-risk and low-risk ACA compared to no ACA dependent on blast counts. Hazard ratios for mortality in imatinib-treated patients with high-risk and low-risk ACA were determined in six different (but overlapping) patient groups (blast increase to 1–30%) together with 95% confidence intervals **a** in peripheral blood and **b** in bone marrow. The size of the circle correlates with the sample size. Thirty-seven patients with high-risk ACA and four patients with low-risk ACA died. In 34 patients with high-risk ACA, causes of death were known. Thirty-two of these (94%) died of progression, including progression-related transplantation in 21 patients. Two patients died of CML-unrelated causes. Causes of death were unknown in three patients. With low-risk ACA, causes of death were CML related in three patients and unknown in one patient.

In the corresponding Cox proportional hazards models (Table 3, Fig. 3), we found an increased hazard to die in the presence of high-risk ACA compared to no ACA with hazard ratios of up to 3.65 (95% CI: 2.32–5.75) in the blood (Fig. 3a) and 6.12 (95% CI: 4.1–9.2) in the marrow (Fig. 3b) when only patients with low blast levels of 1–5% were considered. When restricting the cohorts to larger blast increases, the effect of high-risk ACA on the hazard to die decreased. In the last cohorts of patients with blast increases to at least 20% or 30%, no difference between patients with and without high-risk ACA was found (hazard ratio: 0.83, 95% CI: 0.50–2.89). The hazard ratios for low-risk ACA compared to no ACA were increased to much lesser extents than for high-risk ACA.

### Course of disease and causes of death

Thirty-seven patients with high-risk ACA (41%) died (Flow chart, Fig. 1a, c). The causes of death were known for 34 patients and almost exclusively CML related. Thirty-two patients (94%) died of progression, mostly in BC, including 21 after progression-related transplantation (15 in BC, 1 in AP, 2 after loss of cytogenetic remission, 2 because of no molecular response, and 1 with unknown indication). Two patients died of CML-unrelated causes.

Of 54 (59%) living patients with high-risk ACAs, 21 (23% of total) were transplanted, 8 of these in BC, 2 in AP, 9 because of no molecular response, and 2 with unknown indication. Five (5%) non-transplanted patients are alive after progression (Flow chart, Fig. 1a, c). Twenty-eight patients (31%) are alive without documented progression 0–11 years after emergence of high-risk ACA.

Of the four patients with low-risk ACA who died, three died of CML-related (one after SCT) and one of CML-unrelated causes

## Discussion

We here report that the combination of high-risk ACA and low-level blasts heralds progression and death by CML. This finding has implications for diagnosis and management of patients in end-phase CML. High-risk ACAs allow to identify CML end-phase at an earlier time than is possible with the current blast thresholds [9, 10]. These patients require a change of therapy and/or more intensive treatments, for example, with ponatinib or allogeneic SCT [9]. A clinically relevant, but in 39 patients not statistically significant difference of 30% 2-year survival suggests that outcome of transplanted patients with high-risk ACA depends on disease stage similar to patients without ACA [12]. Successful treatment may explain, at least in part, the plateau phase of survival curves after 4 years.

High-risk ACA at low blast counts are not an indicator of AP as previously reviewed [31, 32], but a marker of progression and death by CML regardless whether they are present at diagnosis or emerge in the course of disease. Sixty-nine percent of patients with high-risk ACA and low blast levels progressed or died of CML during the observation period. This includes transplanted patients who as a rule were transplanted because of progression. Our findings agree with an earlier observation that patients in AP with ACA and blast increase have a worse outcome [33].

Our data suggest that the appropriate time for a change of treatment is emergence of high-risk ACA rather than waiting for an increase of blasts.

High-risk ACA include, in addition to major route ACA (which were defined solely on the basis of their frequency in BC) [1–2], most notably −7/7q−, 3q26.2, and 11q23 rearrangements and complex karyotypes [16–21, 26]. Additional high-risk ACA may surface on continued clinical and cytogenetic scrutiny.

Our definition of high-risk ACA largely agrees with that proposed by others [17, 21], but there are differences. The prognosis with +8 alone, although clearly better than with +8 in combination, is still worse than with low-risk ACA (Fig. 2a). We thus have included +8 alone in the high-risk ACA group. +Ph has an equally unfavorable impact on survival, regardless whether it occurs as a single aberration or in combination (Fig. 2b). In agreement with earlier reports [17, 18], chromosome 19 aberrations exclusively occurred in combination, whereas 3q26.2 was mostly found as a single aberration and rarely at diagnosis [16]. −Y in our analyses had a prognosis not different from no ACA.

Looking at our data in context of the literature [17, 21], a risk stratification of ACA in two risk-groups (high risk with impact on survival and low risk with little or no impact) seems useful for clinical purposes. Due to the limited sample size and the exploratory nature of this work, we would welcome verification of these results by others.

High-risk ACAs were not observed in all BC patients. We cannot rule out that other events such as genetic alterations not detectable by cytogenetics predispose to a blast increase. Gene sequencing detects such alterations [34]. Telomere shortening [35] or increased separase activity [36] may contribute to progression. Likewise, low-risk ACA might include some hitherto unrecognized high-risk ACA as suggested by the three patients with BC and low-risk ACA.

High-risk ACAs were observed in 6% of CP patients, but in 61% of patients who had progressed to BC, whereas low-risk ACAs were observed in 2.1% of CP patients and in 2.8% of patients with BC. This is strong support for a role of high-risk ACA in the evolution of CML and is in line with the hypothesis that BCR-ABL1 predisposes to ACA, which then promote progression. The emergence of high-risk ACA might anticipate and define the point of no return in the evolution of CML indicating non-reversibility by tyrosine kinase inhibitor (TKI). High-risk ACA could emerge as testable cause of non-mutation-related TKI resistance.

A limitation of this study is the follow-up cytogenetics, which have been replaced in many instances by molecular testing increasingly done in the course of the study. Furthermore, most cytogenetic analyses were performed in the first months after diagnosis or when the patients’ conditions were worsening. This might introduce a bias, as patients doing well were usually not analyzed. Also, it is possible that analyses with low numbers of mitoses miss ACA and that the true percentage of patients with ACA is underestimated.

Although the low number of follow-up cytogenetics has limited the direct correlation of ACA with blast increase, it points to the strength of the evidence for the association of high-risk ACA with end-phase CML and survival in the Cox model in spite of missing values.

A correlation with BCR-ABL1 transcript numbers was not conclusive because of too few molecular measurements at early blast increase.

A strength of the study is the size of the cohort as one of the largest of TKI-treated CP-CML patients on whom emergence of ACA and progression to BC were prospectively recorded in parallel over prolonged periods of time. The association of high-risk ACA with progression would not be so obvious in smaller cohorts with shorter observation.

In conclusion, high-risk ACAs are an early marker of CML progression. In the presence of low blast levels, high-risk ACAs indicate death by CML earlier than is possible with standard blast thresholds. An appropriate time for a change of therapy may be emergence of high-risk ACA rather than waiting for an increase of blasts. Cytogenetic monitoring is indicated when signs of progression surface and response to therapy is unsatisfactory.

## Appendix A1. Karyotypes of patients with ACA

**Table:**

No.	Sex	Age^a^	Interval^b^	Karyotype
High-risk ACA in Ph+ cells				
1	F	54	11	46,XX,inv(3)(q21q26),t(7;9;22)(q31;q34;q11) [20]
			16	46,XX,inv(3)(q21q26),t(7;9;22)(q31;q34;q11) [25]
			20	46,XX,inv(3)(q21q26),t(7;9;22)(q31;q34;q11) [25]
2	F	45	28	46,XX,inv(3)(q21q26),t(9;22)(q34;q11) [19]
			32	46,XX,inv(3)(q21q26),t(9;22)(q34;q11) [2]
3	F	61	14	46,XX,t(9;22)(q34;q11) [3]/46,XX,der(7)del(7)(p11)del(7)(q11),t(9;22)(q34;q11) [2]/ 45,XX,der(3;9;17)der(7)del(7)(p11)del(7)(q11),+der(9)ins(9;17)(p11;??),t(9;22)(q34;q11),der(9;17)dic(9;17) (q11;p11)del(9)(p13p24),der(14)t(1;14)(q21;q21),+17,der(22)t(9;22)(q34;q11) [2]/45,X,r(X),der(3;9;17) (3pter?....)der(7)del(7)(p11)del(7)(q11),+der(9)ins(9;17)(p11;??),t(9;22)(q34;q11),der(9;17)dic(9;17)(q11;p11)del(9)(p13p24),der(14)(q21;q21),+17,der(22)t(9;22)(q34;q11) [4]
4	M	42	0	46,XY,inv(3)(p13q25),t(9;22)(q34;q11) [17]
5	F	32	12	46,XX,inv(3)(q21q26),t(9;22)(q34;q11) [15]
6	M	43	8	46,XY,inv(3)(q21q26),t(9;22)(q34;q11) [24]
7	F	37	6	46,XX,t(3;21)(q26;q22),t(9;22)(q34;q11) [15]
			7	46,XX,t(3;21)(q26;q22),t(9;22)(q34;q11) [17]
			8	46,XX,t(3;21)(q26;q22),t(9;22)(q34;q11) [20]
8	M	37	11	46,XY,inv(3)(q21q26),t(9;22)(q34;q11) [9]
			14	46,XY,inv(3)(q21q26),t(9;22)(q34;q11) [3]
			17	46,XY,inv(3)(q21q26),t(9;22)(q34;q11) [2]
			24	46,XY,inv(3)(q21q26),t(9;22)(q34;q11) [3]
			32	46,XY,inv(3)(q21q26),t(9;22)(q34;q11) [2]
			36	46,XY,inv(3)(q21q26),t(9;22)(q34;q11) [2]
			44	46,XY,inv(3)(q21q26),t(9;22)(q34;q11) [6]
			45	46,XY,inv(3)(q21q26),t(9;22)(q34;q11) [20]
9	M	60	6	48–49,XY,t(5;9)(q31;q22),−7,der(7)del(7)(p11)del(7)(q11),+8, der(9)t(7;9)(q11;p23)t(9;22)(q34;q11),+19,der(22)t(9;22)(q34;q11),+der(22)t(9;22)[cp12]
10	M	71	12	46,XY,der(5)t(5;9)(q11;?),−7,der(9)t(9;22)(q34;q11)t(9;9)(p13;?)t(7;9)(?;?), der(9)del(9)(p22)t(9;7)(q?;?)t(5;7)(q?;?),der(22)t(9;22)(q34;q11),+der(22)t(9;22)(q34;q11) [6]
11	M	45	13	46,XY,r(7)(p11q32)del(7)(q11q22),del(9)(p12p24),t(9;22)(q34;q11) [6]
12	M	36	25	43–44,XY,−8,t(9;22)(q34;q11),−11,−15,−17,−18,−19,−20,−22,+2–3mar[cp10]
13	M	44	3	46,XY,t(9;22)(q34;q11) [11]/51,XY,+X,dup(6)(p22p25),+der(6)t(4;6)(q24;q16),t(9;22)(q34;q11),+14,+21, +der(22)t(9;22)(q34;q11) [4]/51,XY,+X,der(4)t(4;8)(p15;q22),dup(6)(p22p25),+der(6)t(4;6)(q24;q16),+14,+21, +der(22)t(9;22)(q34;q11) [2]
14	F	68	10	46,XX,t(9;22)(q34;q11) [6]/46,XX,inv(3)(q21q26),t(9;22)(q34;q11) [6]
			13	46,XX,inv(3)(q21q26),t(9;22)(q34;q11) [2]/45,XX,inv(3)(q21q26),−7,t(9;22)(q34;q11) [10]
15	M	69	9	46,XY,t(9;22)(q34;q11)/48,XY,+8,t(9;22)(q34;q11),+der(22)t(9;22)(q34;q11) [3]/ 50,XY+8,+8,t(9;22)(q34;q11),+19,+der(22)t(9;22)(q34;q11) [4]
			19	45,XY,t(3;12)(q26;q24),−7,t(9;22)(q34;q11) [11]
16	F	54	45	46,XX,t(3;21)(q26;q22),t(9;22)(q34;q11) [25]
			48	46,XX,t(3;21)(q26;q22),t(9;22)(q34;q11) [25]
17	M	64	22	51,XY,+6,+8,+8,der(9)t(9;10)(q34;q22),der(10)t(10;15)(q22;q15), +der(11)t(11;17)(p11.2;q11.2),der(15)t(15;22)(q15;q11.2),der(22)t(9;22)(q34;q11.2),+der(22)t(9;22)(q34;q11.2) [17]
18	M	24	10	45,X,−Y,t(9;22)(q34;q11) [8]/46,X,−Y,t(9;22)(q34;q11.2),+der(22)t(9;22)(q34;q11.2) [4]/ 47,X,−Y,−7,t(9 ;22)(q34;q11.2),+der(22)t(9;22)(q34;q11.2),+mar,+r [8]
19	M	61	0	46,XY,t(9;22)(q34;q11) [14]/46,XY,der(9)t(9;22)(q34;q11),ider(22)(q10)t(9;22)(q34;q11) [11]
20	M	51	44	47,XY,t(9;22)(q34;q11),+der(22)t(9;22)(q34;q11) [4]
21	M	61	46	46,XY,t(9;22)(q34;q11),+der(22)idic(9)(q34)t(9;22)(q34;q11),+der(22)t(9;22)(q34;q11)x3,+mar[cp20]
			64	49,XY,t(9;22)(q34;q11),+der(22)t(9;22)(q34;q11)x3 [3]
22	F	60	2	46,XX,der(7)t(7;22)(q36;q11)del(7)(q11q22),der(9)t(7;9)(q36;q34),der(22)t(9;22)(q34;q11) [21]
23	M	46	0	47,XY,der(9)t(9;22)(q34;q11),del(9)(q33q34),del(22)(q11q12),+der(22)t(9;22)(q34;q11) [25]
24	F	59	6	46,XX,der(9)t(9;22;15)(q34;q11;q26),der(15)t(9;22;15),+22,der(22)idic(22)(p11)t(9;22) [15]
25	M	34	3	48,XY,+X,der(5)t(1;5)(q21;q31),t(9;22;21)(q34;q11;q22),+der(22)t(9;22) [15]
26	M	40	41	47,XY,t(9;22)(q34;q11),+der(22)t(9;22)(q34;q11) [2]
			76	47,XY,t(9;22)(q34;q11),+der(22)t(9;22)(q34;q11) [5]
			78	47,XY,t(9;22)(q34;q11),+der(22)t(9;22)(q34;q11) [8]
			79	47,XY,t(9;22)(q34;q11),+der(22)t(9;22)(q34;q11) [5]
27	M	36	72	46,XY,t(9;22)(q34;q11)/47,XY,t(9;22)(q34;q11),+der(22)t(9;22)(q34;q11) [3]
28	M	54	9	47,XY,t(9;22)(q34;q11),+der(22)t(9;22)(q34;q11) [6]
29	M	44	29	46,XY,t(9;22)(q34;q11) [5]/47,XY,t(9;22)(q34;q11),+der(22)t(9;22)(q34;q11) [11]
			33	46,XY,t(9;22)(q34;q11) [3]/47,XY,t(9;22)(q34;q11),+der(22)t(9;22)(q34;q11) [9]
30	M	32	18	47,XY,t(1;14)(p3?1;q?32),del(9)(p22p24),t(9;22)(q34;q11),+der(22)t(9;22)(q34;q11) [9]/ 46,XY,del(9)(p22p24),t(9;22)(q34;q11),del(12)(p11.2p13) [6]
31	M	78	13	45,X,−Y,del(9)(q22),+der(22)t(9;22)(q34;q11)t(9;9)(q34;q22) [13]
32	M	38	27	47,XY,t(9;22)(q34;q11),+der(22)t(9;22)(q34;q11) [14]
33	M	23	0	46,XY,t(9;22)(q34;q11) [18]/47,idem,+der(22)t(9;22)(q34;q11) [2]
34	M	68	19	46,XX,t(9;22)(q34;q11) [5]/48,XX,t(9;22)(q34;q11),+19,+der(22)t(9;22)(q34;q11) [8]
35	F	69	0	46,XX,t(9;22)(q34;q11) [8]/47,idem,+ider(22)(q10)t(9;22)(q34;q11) [7]
			3	46,XX,t(9;22)(q34;q11) [7]/47,idem,ider(22)(q10)t(9;22)(q34;q11) [3]
36	M	51	27	46,XY,t(9;22)(q34;q11) [14]/48,idem,+19,+der(22)t(9;22)(q34;q11) [2]
37	F	60	35	45,XX,−7,t(9;22)(q34;q11) [7]
			41	46,XX,r(7)(p13q11),t(9;22)(q34;q11) [9]/45,XX,−7,t(9;22)(q34;q11) [4]
			44	46,XX,r(7)(p13q11),t(9;22)(q34;q11) [7]/45,XX,−7,t(9;22)(q34;q11) [2]
38	M	39	12	46,XY,t(9;22)(q34;q11) [7]/47,XY,+8,t(9;22)(q34;q11) [2]/48,XY,+8,t(9;22)(q34;q11),+der(22)t(9;22)(q34;q11) [3]
			88	46,XY,t(9;22)(q34;q11) /47,XY,+8,t(9;22)(q34;q11) /48,XY,+8,t(9;22)(q34;q11),+der(22)t(9;22)(q34;q11) [16]
39	F	58	7	55,XX,+X,+5,+6,+7,+8,t(9;22)(q34;q11),+14,+19,+22,+der(22)t(9;22)(q34;q11)[cp5]
40	M	38	15	47,XY,t(9;22)(q34;q11),+8 [2]
41	M	39	0	47,XY,+8,t(9;22)(q34;q11) [2]/47,XY,+8,t(9;22)(q34;q11),i(17)(q10) [2]/48,XY,+8,t(9;22)(q34;q11),i(17)(q10),+22, del(22)q11) [10]
42	M	64	10	46,XY,t(9;22)(q34;q11) [16]/50,XY,+8,+12,+18,+21,t(9;22)(q34;q11) [3]
43	M	34	5	46,XY,t(9;22)(q34;q11) [13]/47,XY,+8,t(9;22)(q34;q11) [5]
			117	46,XY,t(9;22)(q34;q11) [9]/47,XY,+8,t(9;22)(q34;q11) [6]
44	F	54	73	46,XX,t(9;22)(q34;q11) [2]/47,XX,+8,t(9;22)(q34;q11) [12]
			81	47,XX,+der(8),t(1;8)(q22;p22),t(9;22)(q34;q11) [2]
45	F	42	51	46,XX,t(9;22)(q34;q11) [17]/47,XX,+8,t(9;22)(q34;q11) [3]
46	M	72	0	47,XY,+8,t(9;22)(q34;q11) [20]
			7	46,XY,t(9;22)(q34;q11) [6]/47,XY,+8,t(9;22)(q34;q11) [18]
47	M	40	0	47,XY,+8,t(9;22)(q34;q11) [17]/49,XY,+8,t(9;22)(q34;q11),+19,+20 [7]
48	M	25	0	46,XY,t(9;22)(q34;q11) [22]/47,XY,+8,t(9;22)(q34;q11) [3]
49	F	24	23	46,XX,t(9;22)(q34;q11) [4]/47,XX,+8,t(9;22)(q34;q11) [4]
			29	46,XX,t(9;22)(q34;q11) [11]/47,XX,+8,t(9;22)(q34;q11) [2]
			36	46,XX,t(9;22)(q34;q11) [3]/47,XX,+8,t(9;22)(q34;q11) [18]
			40	47,XX,+8,t(9;22)(q34;q11) [20]
			44	47,XX,+8,t(9;22)(q34;q11) [5]
50	M	31	0	46,XY,t(9;22;10)(q34;q11;p15) [13]/55,XY,+3,+8,t(9;22;10)(q34;q11;p15),+12,+13,+14,+18,+19,+21,+der(22) t(9;22(q34;q11) [7]
51	M	28	0	46,XY,t(9;22)(q34;q11) [10]/47,XY,t(9;22)(q34;q11),+der(22)t(9;22)(q34:q11) [10]
			3	46,XY,t(9;22)(q34;q11) /47,XY,t(9;22)(q34;q11),+der(22)t(9;22)(q34:q11) [20]
52	M	55	0	46,XY,t(9;22)(q34;q11) [7]/49,XY,+8,+10,t(9;22)(q34;q11),+der(22)t(9;22)(q34:q11) [13]
			6	46,XY,t(9;22)(q34;q11) [3]/49,XY,+8,+10,t(9;22)(q34;q11),+der(22)t(9;22)(q34:q11) [12]
53	F	68	0	48,XX,+8,t(9;18)(q34;q21),+der(22)ins(22;9)(q11;34q34) [5]
			6	48,XX,+8,t(9;18)(q34;q21),+der(22)ins(22;9)(q11;34q34) [4]
54	M	38	0	47,XY,+8,t(9;22)(q34;q11) [3]/48,XY,+8,t(9;22)(q34;q11),+der(22)t(9;22)(q34;q11) [11]
55	M	53	0	50,XY,+8,+8,i(17)(q10),+19,+der(22)t(9;22)(q34;q11) [6]
56	M	28	10	45,XY,t(8;21)(q22;q22),t(9;22)(q34;q11),−11/93,XXYY,+8,+8,t(8;21)(q22;q22),t(9;22)(q34;q11),−11 [8]
57	M	70	19	46,XY,t(9;22)(q34;q11) [19]/47,XY,+8,t(9;22)(q34;q11) [4]
			64	46,XY,t(9;22)(q34;q11) [4]/47,XY,+8,t(9;22)(q34;q11) [16]
58	M	55	0	46,XY,t(1;12)(p34;q24),t(1;9;22)(p36;q34;q11) [21]/48,idem,+8,+9 [2]
59	M	46	52	46,XY,t(9;22)(q34;q11) [8]/47,XY,+8,t(9;22)(q34;q11) [7]
			72	46,XY,t(9;22)(q34;q11) [19]/47,XY,+8,t(9;22)(q34;q11) [4]
			80	47,XY,+8,t(9;22)(q34;q11) [4]
			86	46,XY,t(9;22)(q34;q11) [4]/47,XY,+8,t(9;22)(q34;q11) [13]
60	M	64	42	46,XY,t(9;22)(q34;q11) [17]/47,XY,+8,t(9;22)(q34;q11) [2]
			53	46,XY,t(9;22)(q34;q11) [17]/47,XY,+8,t(9;22)(q34;q11) [2]
			57	46,XY,t(9;22)(q34;q11) [5]/47,XY,+8,t(9;22)(q34;q11) [2]
			65	46,XY,t(9;22)(q34;q11) [4]/47,XY,+8,t(9;22)(q34;q11) [2]
			69	47,XY,+8,t(9;22)(q34;q11) [2]/46,XY,del(7)(q11;q22) [6]
61	M	57	0	48,XY,+8,t(9;22)(q34;q11),+19 [25]
62	M	50	76	46,XY,t(9;22)(q34;q11)/47,XY,+8,t(9;22)(q34;q11),idic(17)(p11) [24]
63	M	46	102	47,XY,+8,t(9;22)(q34;q11),i(17)(q10) [6]
64	M	41	0	46,XY,t(9;22)(q34;q11) [10]/44,XY,t(9;22)(q34;q11),−14,i(17)(q10),−18 [15]
65	F	62	10	46,XX,t(1;9;22)(p36;q34;q11) [8]/47,XX,t(1;9;22)(p36;q34;q11),+8 [2]
66	F	76	8	47,XX,+8,t(9;22)(q34;q11) [3]
67	M	44	20	46,XY,t(3;21)(q26;q22),t(9;22)(q34;q11) [16]/46,XY [4]
			34	49,XY,t(3;21)(q26;q22),+8,t(9;22)(q34;q11),+12,+der(22)t(9;22)(q34;q11) [4]/46,XY [19]
68	F	52	0	47,XX,+8,t(9;22)(q34;q11),i(17)(q10) [10]
			3	47,XX,+8,t(9;22)(q34;q11),idic(17)(p12) [4]/46,XX [21]
			8	50,XX,+8,+8,+8,t(9;22)(q34;q11),idic(17)(p12),+19 [7]/46,XX [20]
69	F	56	0	46,XX,t(9;22)(q34;q11) [21]/47,XX,+8,t(9;22)(q34;q11) [2]
70	M	48	0	46,XY,t(9;22)(q34;q11) [6]/47,XY,+8,t(9;22)(q34;q11) [12]
71	M	51	0	46,XY,t(9;22)((q34;q11) [7]/47,XY,+8,t(9;22)(q34;q11),i(17)(q10) [18]
72	F	75	24	47,XX,t(9;22)(q34;q11),+21 [4]
73	F	39	0	46,XX,t(9;22)(q34;q11) [3]/46,idem,t(20;21)(q10;q10) /47,idem,t(20;21)(q10;q10),+21 [11]
74	M	42	19	47,XY,t(9;22)(q34;q11),+17 [20]
			21	47,XY,t(9;22)(q34;q11),+17 [10]
75	F	62	11	46,XX,t(9;11)(p21–22;q23),t(9;22)(q34;q11) [25]
76	M	51	38	46,XY,t(9;22)(q34;q11),inv(16)(p13q22),+der(22)t(9;22)(q34;q11) [3]
77	F	59	32	48,XX,t(9;15;22) (q34;q26;q11),+8,+19 [3]
78	F	51	7	45,XX,der(7;9)(q10;q10)t(9;22)(q34;q11) [8]
79	M	24	8	46,XY,der(9)t(9;22)(q34;q11),der(19)t(9;19)(q34;p13);der(22)t(9;22)(q34;q11) t(9;19)(q34,p13) [2]
80	M	53	0	46,XY,der(9)t(9;22)(q34;q11),idicder(22)(q11)t(9;22)(q34;q11), idicder(22)(q11)t(9;22)(q34;q11) [3]
81	M	55	0	46,XY,der(7)t(7;9)(q11.2;q34),der(9)t(9;22)(q34;q11.2),der(22)t(9;22)(q34;q11.2) t(7;9)(q11.2q34) [13]
82	M	40	0	47,XY,+8,t(9;22)(q34;q11) [16]
83	F	68	13	46,XX,t(3;11)(q26;q23),t(9;22)(q34;q11) [20]
84	F	54	45	46,XX,t(3;21)(q26;q22),t (9;22)(q34;q11) [25]
85		42	40	47,XY,der(8)t(8;9)(p21;p13),+der(8)t(8;9)(p21;p13),del(9)(p13p24), der(9)t(8;9)(p21;p11)t(9;10)(q34;q24),der(10)t(10;22)(q24;q11), t(14;21)(q22;q21),der(22)t(9;22)(q34;q11) [10]
86	M	60	22	46,XY,inv(7)(p22q32),+8,t(9;22)(q34;q11) [20]
87	M	44	0	46,XY,der(9)t(9;10)(q22;q23)t(10;22)(q25;q11),der(10)t(9;10)(q22;q23)t(9;15)(q34;q21),der(15)t(10;15)(q26;q15) t(10;22)(q25;q11),der(22)t(9;22)(q34;q11) [21]
			4	46,XY,der(9)t(9;10)(q22;q23)t(10;22)(q25;q11),der(10)t(9;10)(q22;q23)t(9;15)(q34;q21),der(15)t(10;15)(q26;q15) t(10;22)(q25;q11),der(22)t(9;22)(q34;q11) [8]
			7	46,XY,der(9)t(9;10)(q22;q23)t(10;22)(q25;q11),der(10)t(9;10)(q22;q23)t(9;15)(q34;q21),der(15)t(10;15)(q26;q15) t(10;22)(q25;q11),der(22)t(9;22)(q34;q11) [3]
			10	46,XY,der(9)t(9;10)(q22;q23)t(10;22)(q25;q11),der(10)t(9;10)(q22;q23)t(9;15)(q34;q21),der(15)t(10;15)(q26;q15) t(10;22)(q25;q11),der(22)t(9;22)(q34;q11) [3]
88	F	38	0	46,XX,del(1)(q21),der(9)t(9;22)(q34;q11)t(1;22)(q44;q11),der(22)t(9;22)(q34;q11)t(1;9)(q21;q34) [20]
89	F	66	0	46,XX,del(1)(q32),der(9)t(1;9)(q32;q34)t(1;22)(q44;q11),der(22)t(9;22)(q34;q11) [20]
90	M	55	0	46,XY,t(7;11),del(7q),der(9)t(9;22)(q34;q11),der(11),del(16q),der(17),der(22)t(9;22)(q34;q11) [10]
91	F	47	0	46,XX,t(9;22)(q34;q11) [2]/46,XX,der(7;11)ins(7;11)(p14;p11q25)t(7;11)(p22;p11),t(9;22)(q34;q11) [13]
			3	46,XX,t(9;22)(q34;q11) [5]/45,XX,der(7;11)ins(7;11)(p14;p11q25)t(7;11)(p22;p11),t(9;22)(q34;q11) [14]/ 46,XX,t(9;22)(q34;q11),del(11)(p13) [2]/46,XX,del(4)(q31),der(11)t(4;11)(q?;p13)t(11;13)(q21;q14), der(12)t(11;12)(p13;q24),der(13)t(11;13)(q21;q14) [2]
			6	46,XX,t(9;22)(q34;q11) [2]/45,XX,der(7;11)ins(7;11)(p14;p11q25)t(7;11)(p22;p11),t(9;22)(q34;q11) [9]
Low-risk ACA in Ph+ cells				
92	M	59	0	46,XY,t(9;22)(q34;q11),del(15)(q22),add(17)(p11) [8]
			4	46,XY,t(9;22)(q34;q11),del(15)(q22),add(17)(p11) [7]
			7	46,XY,t(9;22)(q34;q11),del(15)(q22),add(17)(p11) [10]
			10	46,XY,t(9;22)(q34;q11),del(15)(q22),add(17)(p11) [7]
93	M	48	0	46,XY,t(4;6)(q21;p23),t(9;22)(q34;q11) [20]
94	M	36	12	46,XY,t(9;22)(q34;q11) [8]/46,XY,t(9;22)(q34;q11),i(9)(p10),der(17)t(9;17)(q11;p11) [12]
95	F	58	0	46,XX,t(9;22)(q34;q11) [6]/92,XXXX,t(9;22)(q34;q11)x2 [4]
96	M	44	0	46,XY,der(10),t(9;22)(q34;q11) [25]
97	M	46	0	46,XY,t(9;22)(q34;q11),t(14;17)(p11;q11) [20]
98	M	40	0	46,XY,t(9;22)(q34;q11) [2]/46,XY,del(5)(q11q14),t(9;22)(q34;q11) [19]
			6	46,XY,del(5)(q11q14),t(9;22)(q34;q11) [2]
99	F	51	7	46,XX,t(9;22)(q34;q11) [4]/45,XX,der(7;9)(q10;q10)t(9;22)(q34;q11),der(22)t(9;22)(q34;q11) [8]
100	M	27	0	46,XY,t(9;22)(q34;q11) [20]/45,XY,t(9;22)(q34;q11),−21 [4]
101	F	52	0	46,XX,t(2;16)(p2?3;p1?3),t(9;22)(q34;q11) [26]
102	F	61	0	46,XX,t(9;22)(q34;q11) [21]/46,XX,del(6)(q15q23),t(9;22)(q34;q11) [4]
103	F	68	0	46,XX,t(9;22)(q34;q11) /46,XX,del(5)(q13q22),t(9;22)(q34;q11) [24]
104	F	64	0	46,XX,t(5;8)(q14;q23),t(9;22)(q34;q11) [19]
105	M	37	0	46,XY,t(9;22)(q34;q11),t(15;20)(q13;p12) [20]
106	F	45	39	46,XX,t(9;22)(q34;q11.2) [16]/46,XX,t(9;22)(q34;q11.2),add(20)(p11.2) [9]
107	M	19	0	46,XY,der(1)t(1;9)(q21;q34)t(9;22)(q34:q11),der(9)t(1;9)(q21;q34)t(9;22)(q34:q11),der(22)t(9;22)(q34;q11) [3]
108	F	65	86	46,XX,t(9;22)(q34;q11) [17]/46,XX,del(X)(p?21),t(9;22)(q34;q11) [6]
109	M	64	10	46,XY,add(9)(q34),add(9)(q32–34),der(22)t(9;22)(q34;q11) [10]
			14	46,XY,t(1;3)(p36;q2?6),add(9)(q34),add(9)(q32–34),der(22)t(9;22)(q34;q11) [10]
110	F	56	0	46,XX,t(1;21)(q21;q22),t(9;22)(q34;q11) [20]
			12	46,XX,t(1;21)(q21;q22),t(9;22)(q34;q11) [15]
			24	46,XX,t(1;21)(q21;q22),t(9;22)(q34;q11) [2]
111	M	36	0	46,XY,t(9;22)(q34;q11),t(11;19)(q14.1;q13) [20]
112	M	62	0	46,XY,t(9;22)(q34;q11) [2]/46,idem,add(8)(q24) [2]/45,idem,der(18)t(10;18)(q11;p11) [10]
113	M	61	0	46,XY,t(1;9)(q24;q31),t(9;22)(q34;q11) [20]
114	F	46	0	46,XX,der(2)t(2;4)(q37;q21)del(4)(q21),t(9;22)(q34;q11) [20]
115	M	70	0	46,XY,der(9)t(9;22)(q34;q11),t(10;22)(q25;q13) [17]
			4	46,XY,der(9)t(9;22)(q34;q11),t(10;22)(q25;q13) [3]
			7	45,X,−Y [3]/46,XY,der(9)t(9;22)(q34;q11),t(10;22)(q25;q13) [2]
116	F	69	94	46,XX,t(9;22)(q34;q11),?del(17)(p12) [4]
117	F	49	0	46,XX,del(3)(p11p2?1)or(p21),t(9;22)(q34;q11) [12]/46,XX,del(3)(p11p2?1)or(p21),del(5)(q15q31),t(9;22)(q34;q11) /46,XX,t(9;22)(q34;q11) [2]
118	F	76	0	46,XX,t(9;22)(q34;q11) [7]/46,XX,t(9;22)(q34;q11),der(19)t(19;?)(p13.3;?) [13]
			4	46,XX,t(9;22)(q34;q11) [7]/46,XX,t(9;22)(q34;q11),der(19)t(17;19)(q22;p13) [13]
119	M	40	3	46,XY,t(6;15),t(9;22)(q34;q11) [6]
120	M	66	11	46,XY,t(9;22)(q34;q11) [9]/46,XY,der(6)t(6;17)(p21;q11),t(9;22)(q34;q11) [11]
121	F	43	0	46,XX,t(7;7)(p22;q22),t(9;22;9)(q34;q11;p24) [16]
			60	40–43,XX,t(7;7)(p22;q22),t(9;22;9)(q34;q11;p24),inc [cp3]
122	M	24	6	46,XY,t(2;12)(q33;p13),t(9;22)(q34;q11) [20]
123	F	31	11	46,XX,t(9;22)(q34;q11) [18]/ 46,XX,t(9;22)(q34;q11),ins(11;11)(p15;p11.2p13) [4]
			25	46,XX,t(9;22)(q34;q11) [9]/ 46,XX,t(9;22)(q34;q11),ins(11;11)(p15;p11.2p13) [5]

^a^Age at diagnosis (years).

^b^Interval between diagnosis and emergence of ACA (months).
